# Supplementary figures and images for: Dynamic prediction of carbon prices based on the multi-frequency combined model
Source: PeerJ Comput Sci. 2025 Apr 17;11:e2827. doi: 10.7717/peerj-cs.2827 (PMC12190694; doi:10.7717/peerj-cs.2827)

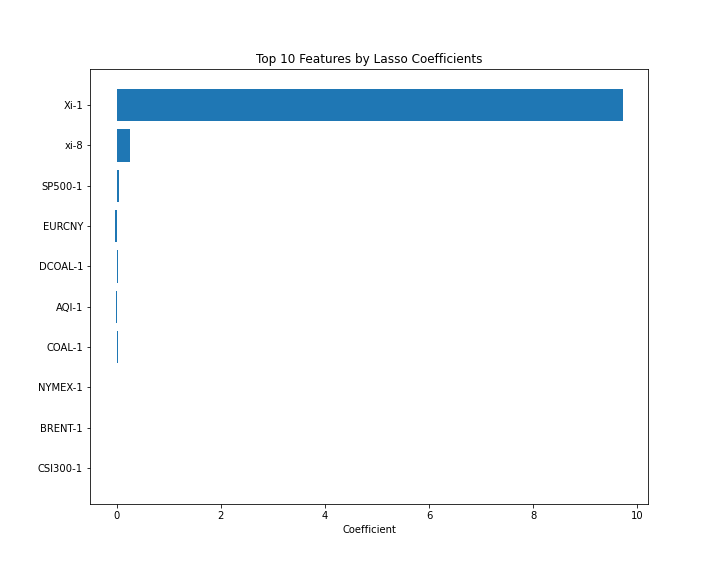

Supplement: Supplemental Information 1 [file peerj-cs-11-2827-s001.zip › Source code/2 lasso/top_10_features_importance.png]

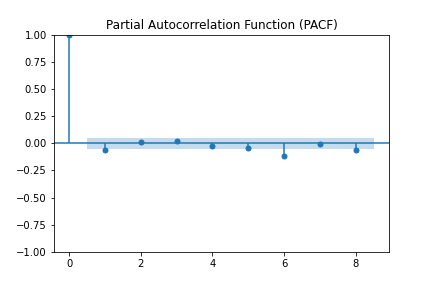

Supplement: Supplemental Information 1 [file peerj-cs-11-2827-s001.zip › Source code/3 PACF/pacf_plot.png]

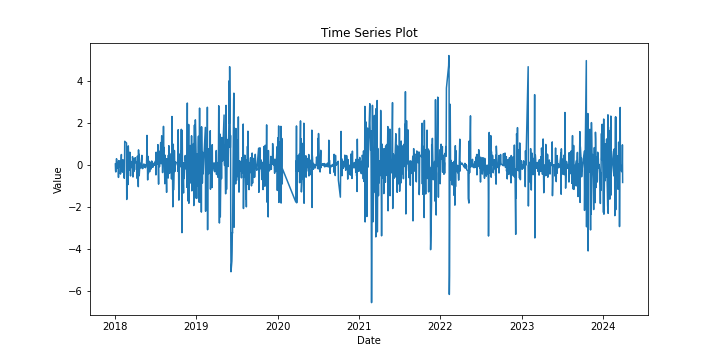

Supplement: Supplemental Information 1 [file peerj-cs-11-2827-s001.zip › Source code/3 PACF/time_series_plot.png]

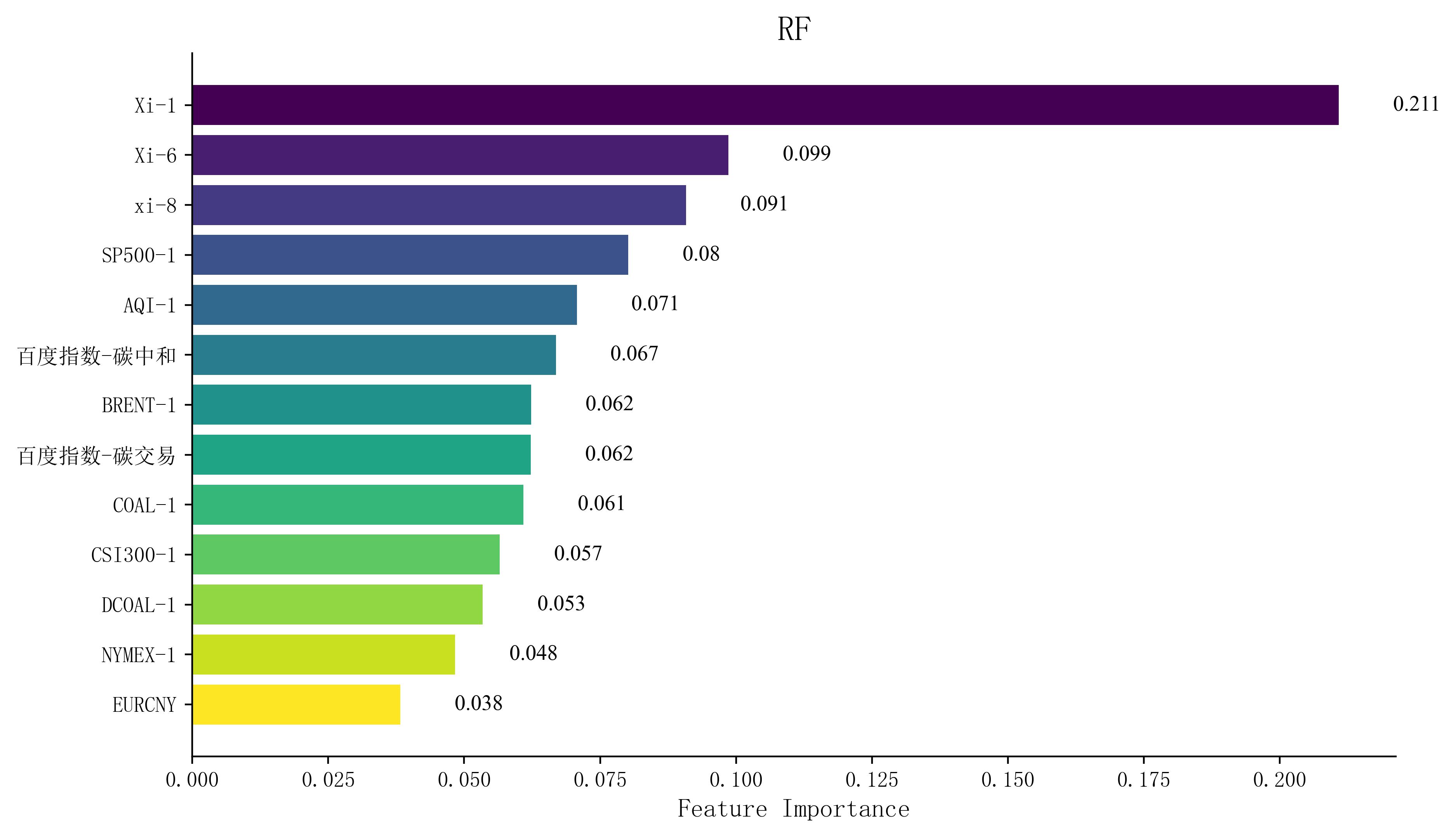

Supplement: Supplemental Information 1 [file peerj-cs-11-2827-s001.zip › Source code/7Characteristic importance analysis/RF Feature Importance.jpg]

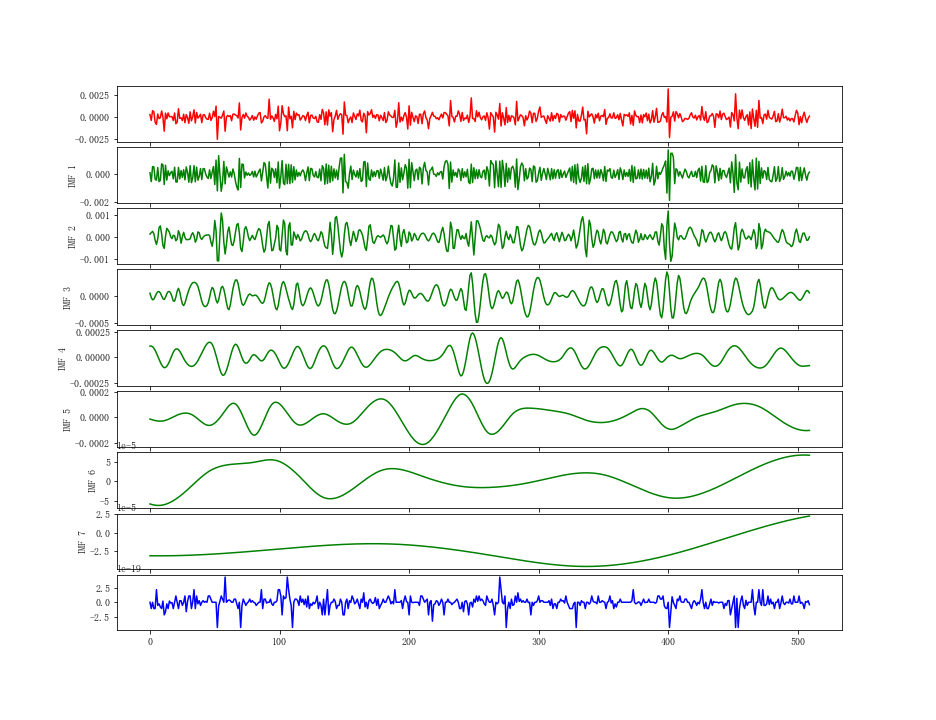

Supplement: Supplemental Information 1 [file peerj-cs-11-2827-s001.zip › Source code/comparison experiment:COA-XGBoost-CEEMDAN/wl_res.png]
